# E3 ligase RNF145 regulates airway smooth muscle cell proliferation *via* the PP2A-p70S6K signaling axis

**DOI:** 10.1016/j.jbc.2026.113427

**Published:** 2026-08-10

**Authors:** Vijaya Kumar Gangipangi, Erin O. Curley, Deepak A. Deshpande, Pawan Sharma

**Affiliations:** 1 Department of Pathology and Laboratory Medicine, Thoracic Medicine and Surgery, Center for Inflammation & Lung Research, Aging+Cardiovascular Discovery Centre, Lewis Katz School of Medicine, Temple University, Philadelphia, Pennsylvania, USA; 2 Center for Translational Medicine, Thomas Jefferson University, Philadelphia, Pennsylvania, USA

**Keywords:** E3 ubiquitin ligase, airway remodeling, airway smooth muscle, cell proliferation

## Abstract

Airway smooth muscle (ASM) is a key determinant of airway caliber and a major contributor to structural remodeling in obstructive lung diseases, including asthma and COPD. ASM proliferation is regulated by transcriptional and post-transcriptional mechanisms, including ubiquitin-dependent protein turnover mediated by E3 ubiquitin ligases. Here, we investigated the role of the RING-type E3 ligase RNF145 in mitogenic signaling and ASM cell proliferation. Human ASM cells were transfected with RNF145 shRNA or treated with the E3 ligase inhibitor SMER3, followed by stimulation with fetal bovine serum (FBS) or platelet-derived growth factor (PDGF). RNF145 knockdown or SMER3 treatment dose-dependently inhibited mitogen-induced ASM cell proliferation without inducing cytotoxicity, supporting a pro-mitogenic role for RNF145. Both interventions reduced phosphorylation of p70S6K at Thr421/Ser424 and Thr389 without affecting ERK MAPK signaling. Inhibition of PP2A and PP1 with okadaic acid or calyculin A restored p70S6K phosphorylation in RNF145-deficient cells, while SMER3 increased serine/threonine phosphatase activity. Mechanistically, RNF145 promoted K48-linked ubiquitination and proteasomal degradation of the PP2A scaffold subunit Aα, thereby limiting PP2A holoenzyme assembly during mitogenic signaling. Re-expression of RNF145 in RNF145-deficient cells reduced PP2A Aα abundance and restored p70S6K activation and ASM cell proliferation. These findings identify RNF145 as a positive regulator of ASM cell proliferation that sustains p70S6K signaling by suppressing PP2A. The RNF145–PP2A–p70S6K axis may therefore represent a therapeutic target for airway remodeling in chronic obstructive airway diseases.

Airway smooth muscle (ASM) maintains airway tone under physiological conditions and is a key determinant of lung structure and function (1). Excessive ASM cell proliferation is a hallmark of asthma and chronic obstructive pulmonary disease (COPD), the two most common obstructive airway diseases worldwide (2). In asthma, increased ASM mass contributes to airflow limitation, disease severity, and reduced responsiveness to bronchodilator and anti-inflammatory therapies (2, 3, 4). Current asthma medications are ineffective in controlling ASM remodeling (5). Defining novel molecular pathways that control ASM proliferation may uncover new targets to limit airway remodeling and improve clinical outcomes in chronic airway diseases.

The proliferation of ASM cells is regulated by complex transcriptional and post-transcriptional mechanisms, including protein turnover mediated by ubiquitination. Ubiquitination is a post-translational modification that regulates numerous cellular processes, including intracellular signaling, cell proliferation, and protein degradation (6, 7, 8, 9). This process is mediated by a cascade of three classes of enzymes: an E1 ubiquitin-activating enzyme, E2 conjugating enzymes, and substrate-specific E3 ubiquitin ligases. In mammals, the ubiquitin system consists of two E1 enzymes, ∼40 E2 enzymes, and more than 600 E3 ligases (10). Ubiquitin activation by the E1 enzyme requires ATP, after which ubiquitin is transferred to an E2 enzyme, and an E3 ligase then catalyzes its attachment to the target substrate (11). Ubiquitin can be linked to substrates through seven lysine residues (K-6, -11, −27, −29, −33, −48, −63) or the N-terminal methionine (M1). Among these, K48- and K63-linked ubiquitination are the most common and well-characterized in eukaryotic cells, each mediating distinct biological outcomes (11). The diversity of linkage types enables precise regulation of protein fate and signaling/functional outcomes. Furthermore, E3 ligases confer substrate specificity and hence play a pivotal role in regulating protein levels. Based on their mechanisms, E3 ligases are broadly categorized into four types: HECT (Homologous to E6AP C Terminus), RBR (RING-between-RING), RING (Really Interesting New Gene), and U-box. HECT and RBR ligases form a thioester intermediate with ubiquitin before transfer, whereas RING and U-box ligases mediate direct ubiquitin transfer from E2 to substrate. RING-type E3 ligases, the largest and most diverse family (12, 13), regulate proliferation, migration, apoptosis, and metabolism across multiple cancers (14, 15, 16, 17). RNF145 is an ER-localized RING-type E3 ligase with 14 transmembrane domains and a catalytic RING finger domain (18, 19).

Elevated RNF145 expression promotes proliferation in oral, liver, and ovarian cancers (20, 21, 22), while emerging evidence links E3 ligases to asthma pathogenesis through their modulation of immune and inflammatory pathways (23, 24). In addition, genome-wide association studies have identified RNF145 (rs10076782) as a locus associated with respiratory symptoms, lung growth, and pulmonary function (25). However, its role in airway cellular function remains undefined. Given the central role of ASM in normal lung physiology and in the pathology of obstructive airway diseases, we hypothesized that RNF145 regulates ASM cell proliferation and sought to define the molecular mechanism by which RNF145 controls pro-mitogenic signaling in ASM cells. Our data show that RNF145 suppresses PP2A subunit Aα, the structural subunit of PP2A, through K48-linked ubiquitin-mediated degradation, thereby reducing PP2A activity and sustaining p70S6K phosphorylation, a key mediator of mitogenic signaling in ASM cells. Together, these findings identify the RNF145-PP2A-p70S6K axis as a potential therapeutic target for limiting airway remodeling in asthma.

## Results

### RNF145 inhibition by SMER3 and shRNA-mediated gene knockdown reduces mitogen-induced human ASM cell proliferation

**Figure 1:**
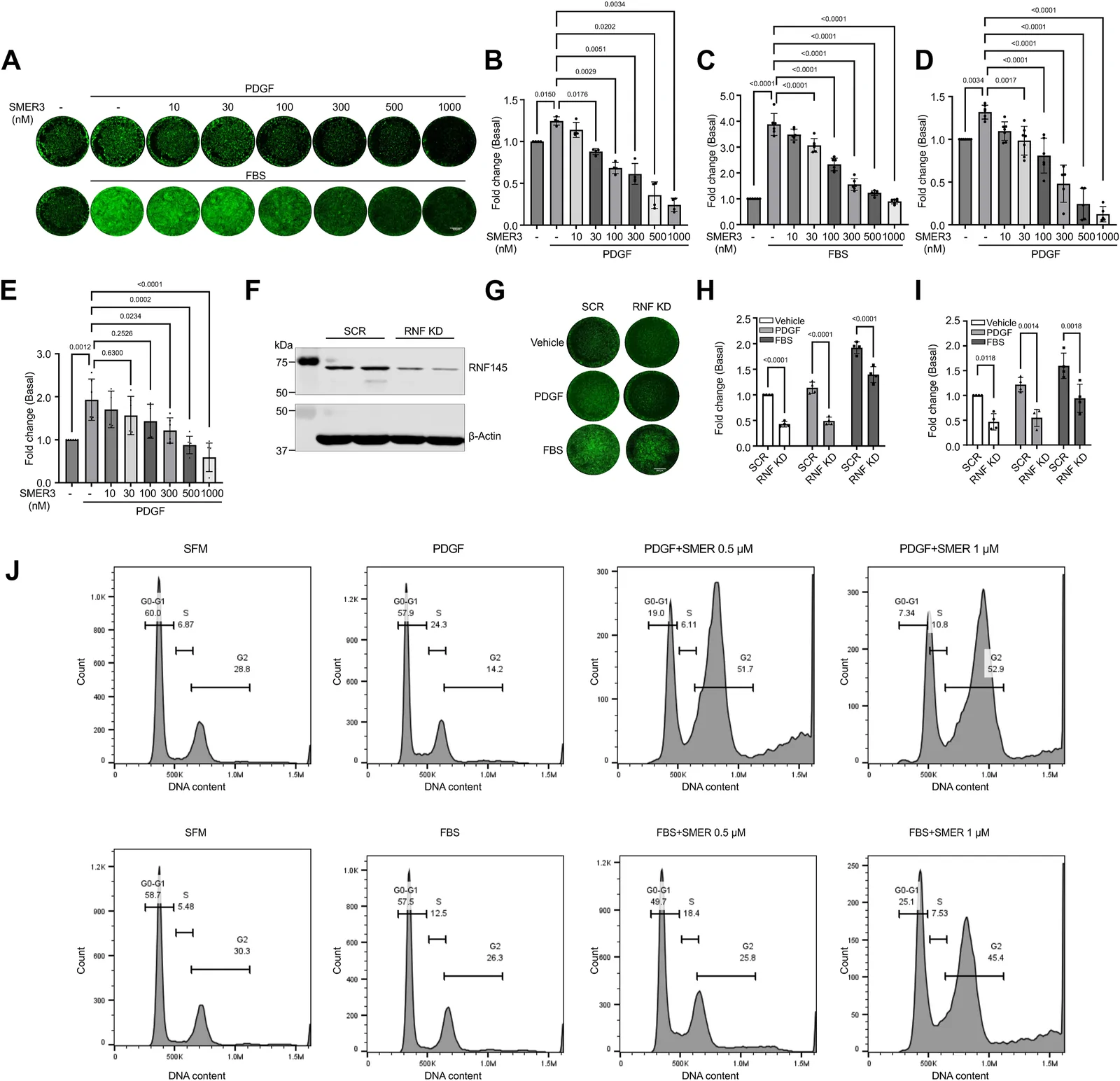
**RNF145 inhibition by SMER3 or shRNA reduces mitogen-induced proliferation of human ASM cells**. Human ASM cells were treated with SMER3 (10–1000 nM) or vehicle for 30 min or subjected to shRNA-mediated RNF145 knockdown (RNF KD), followed by stimulation with PDGF (10 ng/ml) or FBS (10%) for 72 h. Cell proliferation was assessed by CyQuant and Alamar Blue assays. *A*, representative CyQuant fluorescence images. B, C, quantification of CyQuant fluorescence intensity as a measure of DNA content. *D, E*, alamar Blue fluorescence intensity. *F*, immunoblot confirming RNF145 knockdown; β-actin was used as a loading control. *G*, representative CyQuant images of RNF KD cells. *H*, quantification of CyQuant fluorescence intensity. *I*, alamar Blue fluorescence intensity. *J*, human ASM cells were treated with SMER3, stained with propidium iodide (PI), and analyzed by flow cytometry. Representative DNA content histograms and corresponding quantification of cell cycle distribution across the G0/G1, S, and G2/M phases are shown. Statistical analyses were performed using one-way or two-way ANOVA followed by Tukey's or Bonferroni’s multiple-comparisons test. Exact *p* values are indicated in the figures. A *p* value < 0.05 was considered statistically significant.

To define the role of RNF145, a RING-type E3 ligase, in ASM cell proliferation, we employed both pharmacological and molecular knockdown approaches. Cells were treated with SMER3 (10, 30, 100, 300, 500, and 1000 nM) or vehicle or transduced with shRNA targeting RNF145 (RNF KD), and cell proliferation upon treatment with PDGF (10 ng/ml) or 10% FBS for 72 h was assessed using the CyQuant and Alamar Blue assays. SMER3 treatment resulted in a dose-dependent reduction in mitogen-induced ASM cell proliferation (Fig. 1, *A*–*E*). SMER3 treatment attenuated PDGF- or FBS-induced cell proliferation by 45 to 80% and 40 to 77%, respectively, as assessed using the CyQuant assay (Fig. 1, *B* and *C*). The Alamar Blue assay revealed a 38% to 90% and 26% to 70% attenuation of PDGF- or FBS-induced cell proliferation by SMER3, respectively (Fig. 1, *D* and *E*). Furthermore, we assessed the effect of SMER3 on cell cycle regulation by flow cytometry following propidium iodide staining. Representative histograms revealed the distribution of ASM cells across the G0/G1, S, and G2/M phases. Under mitogen-stimulated conditions (PDGF/FBS), ASM cells showed enhanced cell-cycle progression, consistent with increased cell proliferation. In contrast, treatment with SMER3 (500 nM and 1 μM) caused accumulation of cells in the G2/M phase, with a corresponding reduction in the S-phase population (Fig. 1*J*), consistent with G2/M cell cycle arrest and reduced proliferation.

RNF KD in human ASM cells led to a significant reduction in mitogen-induced cell proliferation (Fig. 1, *G*–*I*). RNF145 knockdown was confirmed by immunoblotting (Fig. 1*F*). Quantitative fluorescence intensity analysis showed that RNF KD reduced PDGF- and FBS-induced cell proliferation by 56% and 30%, respectively (Fig. 1, *G*–*H*) in the CyQuant assay and by 54% and 41% in the Alamar Blue assay (Fig. 1, *I*). These results collectively demonstrate that pharmacological or genetic inhibition of RNF145 suppresses ASM cell proliferation and suggest a pivotal role for RNF145 in regulating ASM cell proliferation.

### RNF145 inhibition reduces the mitogen-induced phosphorylation of p70S6 kinase in ASM cells

**Figure 2:**
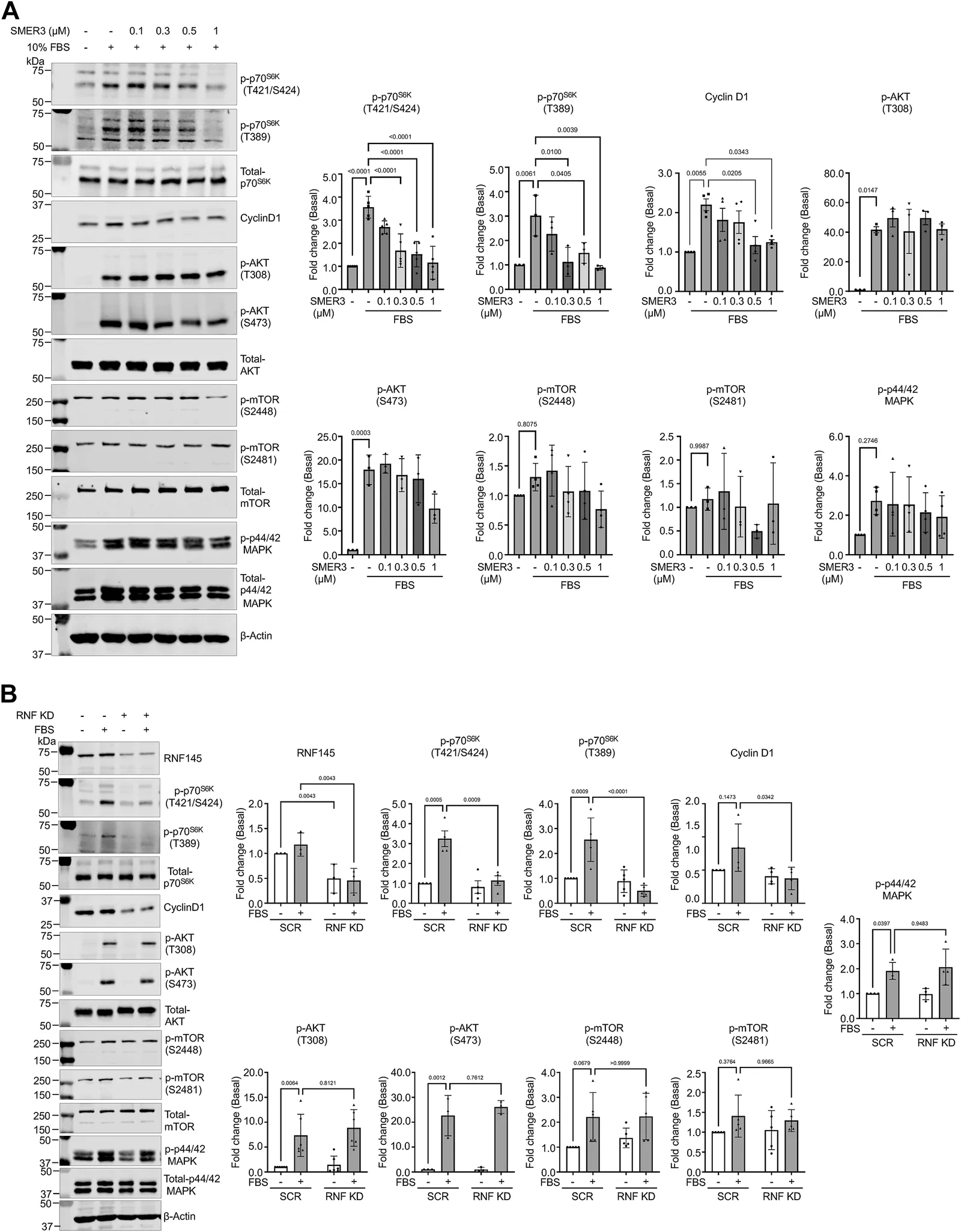

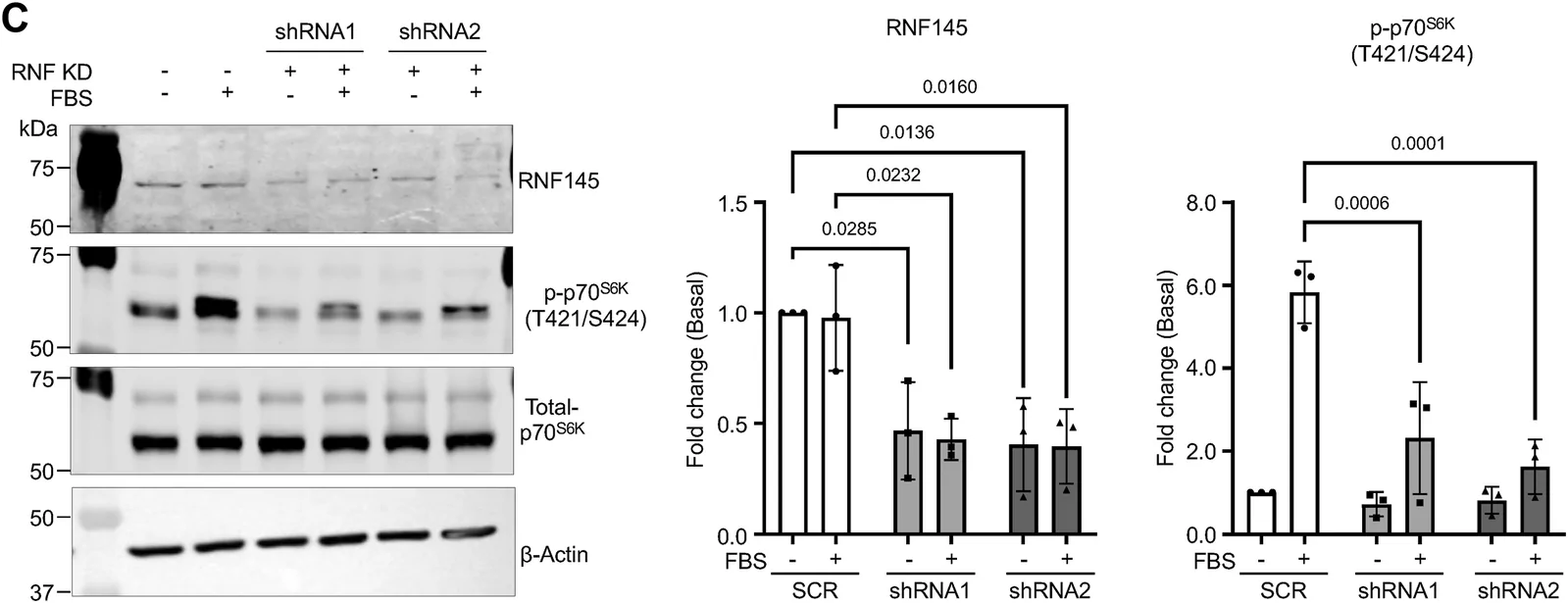
**RNF145 inhibition with SMER3 or shRNA-mediated knockdown reduces mitogen-induced phosphorylation of p70S6K in ASM cells.** Human ASM cells were pretreated with SMER3 (0, 100, 300, 500, or 1000 nM) for 30 min or subjected to shRNA-mediated RNF145 knockdown (RNF KD), followed by stimulation with FBS (10%) for 30 min. Cell lysates were immunoblotted for RNF145, phosphorylated p70S6K (T421/S424 and T389), cyclin D1, p-AKT (T308 and S473), p-mTOR (S2448 and S2481), p-p44/42 MAPK, total p70S6K, AKT, mTOR, p44/42 MAPK, and β-actin. Phosphorylated proteins were normalized to their corresponding total proteins, and β-actin was used as a loading control. *A*, Representative immunoblots from SMER3-treated cells. *B*, Representative immunoblots from RNF KD cells. *C*, representative immunoblots from cells transduced with two independent shRNAs targeting RNF145. Quantitative densitometric analyses are shown. Statistical analyses were performed using one-way or two-way ANOVA followed by Tukey’s multiple-comparisons test, as appropriate. Exact *p* values are indicated in the figures. A *p* value < 0.05 was considered statistically significant.

Next, to determine the molecular mechanism by which RNF145 inhibition reduces ASM cell proliferation, we assessed the effect of RNF145 inhibition on pro-mitogenic signaling pathways associated with ASM cell proliferation, such as MAP kinase and PI3K-AKT signaling (26, 27, 28). Human ASM cells were pretreated with SMER3 (100, 300, 500, and 1000 nM) for 30 min, followed by stimulation with 10% FBS for 30 min, and then the phosphorylation of AKT, mTOR, p70S6K, and p44/42 MAPK was assessed by immunoblotting using cell lysates. FBS treatment increased phosphorylation of p44/42 MAPK, AKT, mTOR, and p70S6K in human ASM cells (Fig. 2*A*). SMER3 pre-treatment attenuated FBS-induced phosphorylation of p70S6K at the T421/S424 and T389 sites in a dose-dependent manner (Fig. 2*A*). Interestingly, SMER3 did not affect the phosphorylation of AKT or mTOR, upstream kinases of p70S6K in the AKT signaling pathway nor did it alter p44/42 MAPK phosphorylation, an effector kinase of the MAPK signaling cascade (Fig. 2*A*).

Next, we examined the impact of RNF KD on PI3K-AKT and MAPK signaling. Western blot analysis showed that RNF KD using two different shRNA constructs markedly reduced FBS-induced phosphorylation of p70S6K at both T421/S424 and T389 by 60.3% and 70.1%, respectively (Fig. 2, *B* and *C*). Similar to SMER3 treatment, RNF KD did not affect the phosphorylation of AKT or mTOR, the upstream kinases of p70S6K in the AKT signaling pathway, or p44/42 MAPK phosphorylation, an effector kinase in the MAPK cascade (Fig. 2, *A* and *B*). Next, we assessed the effect of RNF KD and SMER3 on cyclin D1 expression, a marker of cell proliferation downstream of promitogenic signaling. RNF KD and SMER3 treatment attenuated mitogen-induced cyclin D1 expression (Fig. 2, *A* and *B*).

Collectively, these findings suggest that the reduced ASM proliferation observed following SMER3 treatment or RNF145 knockdown results specifically from diminished p70S6K phosphorylation, without affecting other canonical mitogenic signaling pathways.

### RNF145 regulates p70S6K activation through serine/threonine protein phosphatases (PP2A and PP1) in ASM cells

**Figure 3:**
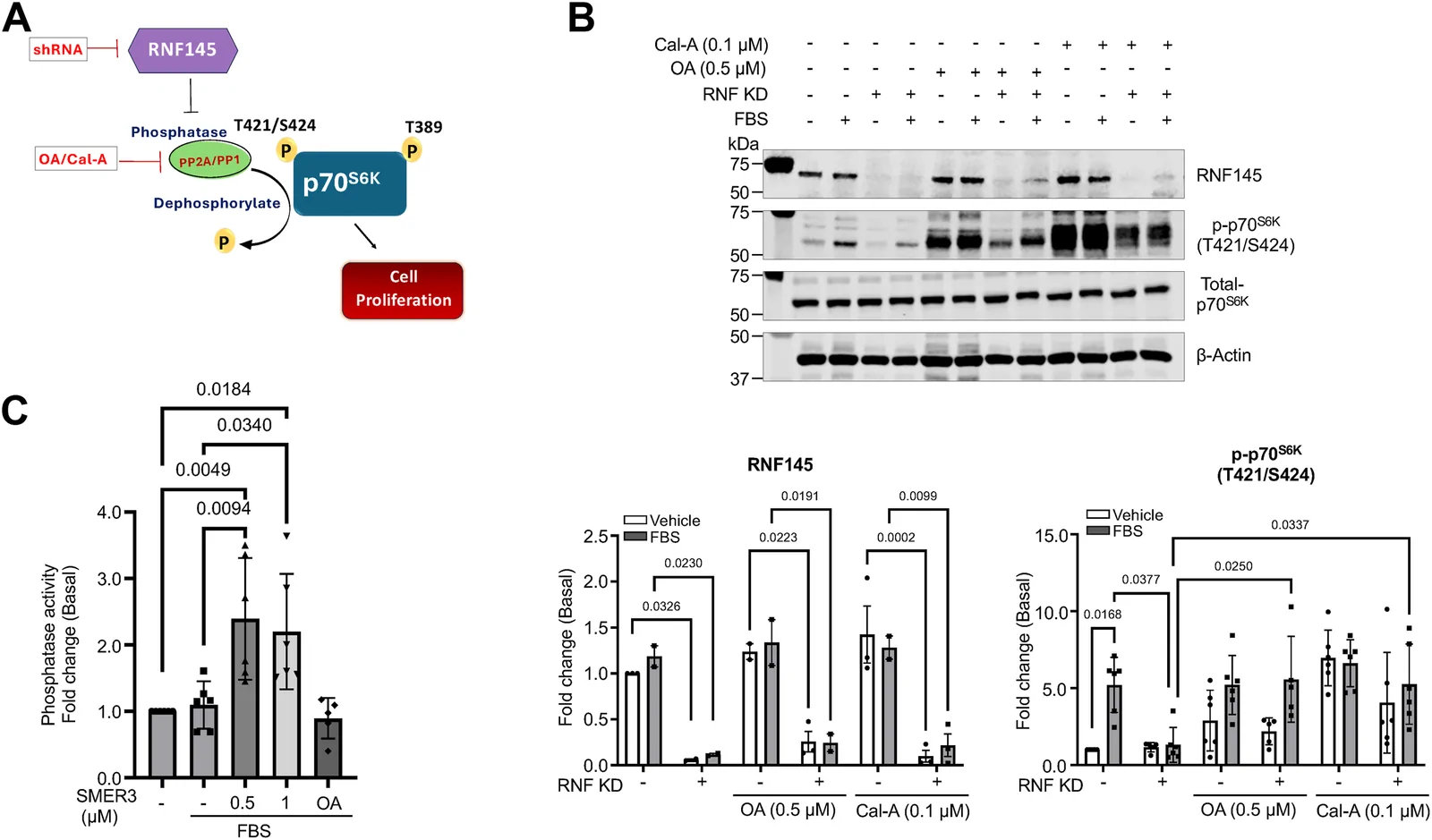
**RNF145 regulates p70S6K activation via serine/threonine protein phosphatases in ASM cells**. *A*, schematic of RNF145-mediated regulation of p70S6K activation through serine/threonine phosphatases. *B*, human ASM cells were subjected to RNF145 shRNA knockdown (RNF KD) and pretreated with the serine/threonine phosphatase inhibitors okadaic acid (OA, 500 nM) or calyculin A (Cal-A, 100 nM) for 30 min, followed by stimulation with FBS (10%) for 30 min. Cell lysates were immunoblotted for RNF145, p-p70S6K (T421/S424), total p70S6K, AKT, mTOR, p44/42 MAPK, and β-actin. Representative immunoblots and quantitative densitometric analyses of RNF145 and p-p70S6K (T421/S424) are shown. *C*, human ASM cells were treated with SMER3 (0.5 or 1 μM), and serine/threonine phosphatase activity was assessed using the K-R-pT-I-R-R substrate. Okadaic acid (OA, 500 nM) was used as an inhibition control. Quantitative analysis of phosphatase activity is shown. Statistical analyses were performed using one-way or two-way ANOVA followed by Tukey’s or Šídák’s multiple-comparisons test, as appropriate. Exact *p* values are indicated in the figures. A *p* value < 0.05 was considered statistically significant.

To gain a deeper understanding of RNF145's role in regulating p70S6 kinase activation, we hypothesized that RNF145 regulates p70S6K phosphorylation by modulating the levels of serine/threonine protein phosphatases (Fig. 3*A*). To test this, RNF145-knocked down human ASM cells were pretreated with pan serine/threonine phosphatase inhibitors okadaic acid (OA, 500 nM) and calyculin A (Cal-A, 100 nM) for 30 min, then stimulated with 10% FBS for 30 min. Cell lysates were used to determine p70S6K phosphorylation by immunoblotting. RNF KD attenuated FBS-induced p70S6K phosphorylation. Pretreatment with OA and Cal-A significantly reversed this reduction (Fig. 3*B*). Together, these results suggest that loss of RNF145 enhances PP2A- and PP1-dependent phosphatase activity, thereby promoting p70S6K dephosphorylation in response to mitogenic stimulation.

### RNF145 regulates PP2A subunit Aα turnover during mitogen stimulation

**Figure 4:**
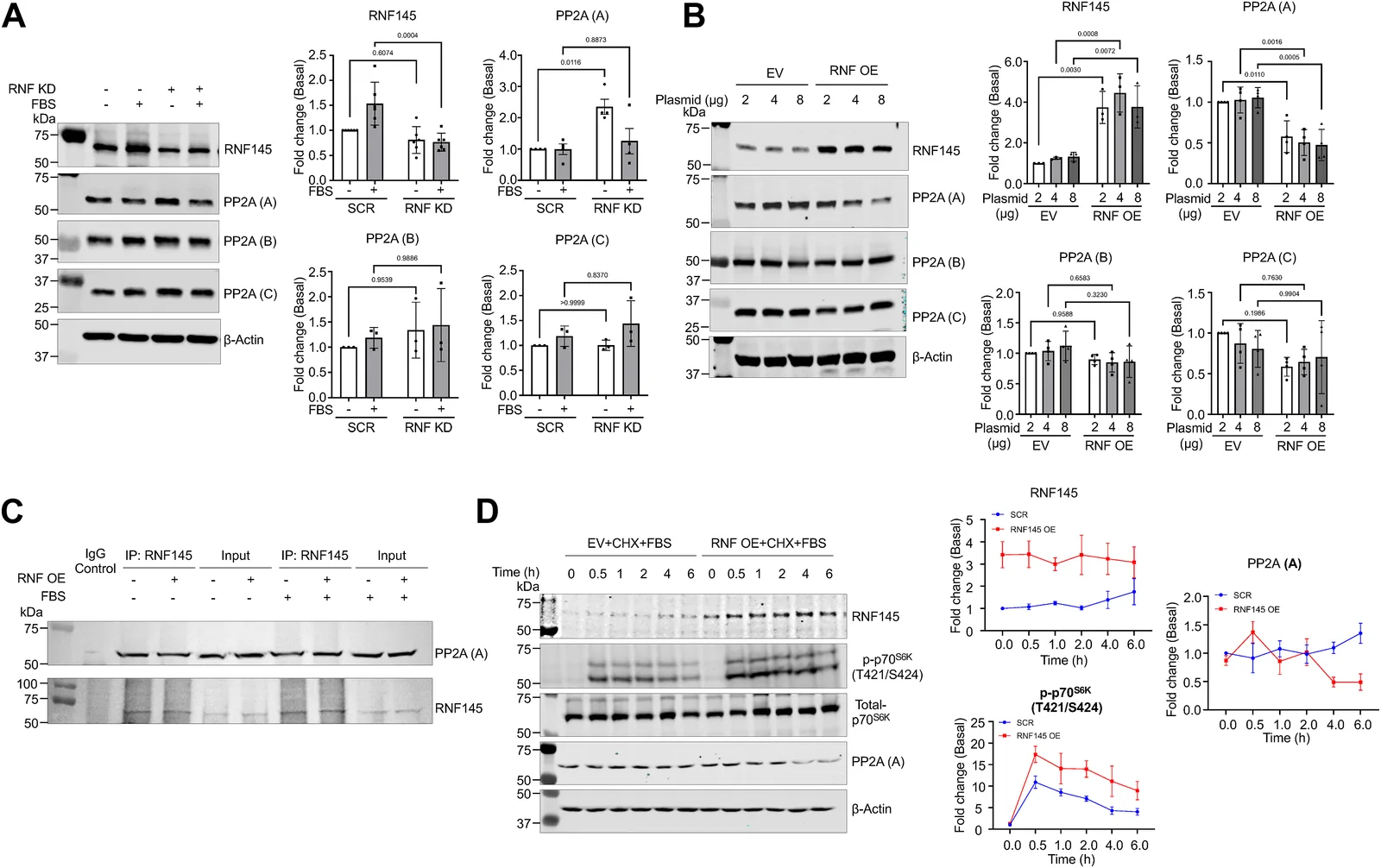
**RNF145 regulates PP2A subunit A**α **turnover during mitogenic stimulation.***A*, Human ASM cells were subjected to RNF145 shRNA knockdown and stimulated with FBS (10%) for 30 min. Cell lysates were immunoblotted for RNF145, PP2A subunits A, B, and C, and β-actin. Representative immunoblots and quantitative densitometric analyses of each protein are shown. *B*, HEK293 cells were transfected with increasing amounts of RNF145 plasmid (RNF OE; 2, 4, or 8 μg). Lysates were immunoblotted for RNF145, PP2A subunits A, B, and C, and β-actin. Representative immunoblots and quantitative densitometric analyses are shown. *C*, RNF145 was immunoprecipitated from RNF OE HEK293 cells using protein A magnetic beads, followed by immunoblotting for PP2A subunits Aα. Representative immunoblots are shown. *D*, RNF OE HEK293 cells were pretreated with cycloheximide (CHX, 10 μg/ml) for 30 min to inhibit protein synthesis, followed by stimulation with FBS (10%) for 0, 0.5, 1, 2, 4, or 6 h. Lysates were immunoblotted for RNF145, p-p70S6K (T421/S424), total p70S6K, PP2A subunit Aα, and β-actin. Representative immunoblots and quantitative densitometric analyses are shown. Statistical analyses were performed using two-way ANOVA followed by Tukey’s or Šídák’s multiple-comparisons test, as appropriate. Exact *p* values are indicated in the figures. A *p* value < 0.05 was considered statistically significant.

Next, we assessed the phosphatase activity in cells treated with SMER3. Pharmacologic inhibition of RNF145 with SMER3 significantly increased serine/threonine phosphatase activity (Fig. 3*C*). We further investigated the specific phosphatases that RNF145 regulates. Previous studies have shown that p70S6K activity is controlled by phosphatases PP1 and PP2A (29, 30, 31, 32). To determine the involvement of PP1 and PP2A in RNF145-mediated p70S6 kinase regulation, we assessed the levels of PP1α and PP2A in RNF KD human ASM cells and RNF145-overexpressing HEK cells (RNF OE). Western blot analysis revealed that RNF KD significantly upregulated PP2A subunit Aα expression, with protein levels approximately 1.7-fold higher compared to scrambled shRNA-transfected cells (Fig. 4*A*). Conversely, RNF OE markedly reduced PP2A subunit Aα expression compared with vector-transfected cells (Fig. 4*B*). Furthermore, RNF145 knockdown in human ASM cells or RNF145 overexpression in HEK293 cells did not change the expression of PP2A subunits B and C (Fig. 4, *A* and *B*). These findings suggest that RNF145 regulates the turnover of PP2A subunit Aα.

Next, we assessed the association between RNF145 and the PP2A structural subunit Aα using co-immunoprecipitation. RNF145 was immunoprecipitated using cell lysates obtained from HEK293 cells transfected with empty vector (EV) or RNF145 overexpression (RNF OE) constructs, with or without fetal bovine serum (FBS) stimulation, and the precipitates were probed for PP2A subunit Aα by immunoblotting. A strong PP2A subunit Aα signal was detected in RNF OE samples compared with empty vector controls (Fig. 4*C*), suggesting an interaction between PP2A and RNF145. This interaction was further enhanced following FBS stimulation. These results demonstrate that RNF145 associates with PP2A subunit Aα and that their association is dynamically regulated by mitogen.

We further investigated the time-dependent effect of RNF145 overexpression on p70S6K signaling and PP2A subunit Aα protein stability by conducting cycloheximide chase experiments in RNF145-overexpressing HEK293 cells. Cells were pre-treated with cycloheximide (Thermo Scientific, #J66901.03) for 30 min to inhibit new protein synthesis, followed by stimulation with FBS, and lysates were harvested at 0.5, 1, 2, 4, and 6 h. In empty vector-transfected cells, FBS treatment showed a rapid decline in p70S6K phosphorylation, with a 63% reduction by 6 h (Fig. 4*D*). In the cells overexpressing RNF145, FBS-induced p70S6K phosphorylation was maintained at relatively high levels throughout the 6-h time course. Notably, the sustained phosphorylation of p70S6K in RNF145-overexpressing cells coincided with a marked reduction of PP2A subunit Aα protein levels (Fig. 4*D*). In cells transfected with an empty vector, PP2A subunit Aα expression was higher than in RNF145-transfected cells (Fig. 4*D*). Taken together, our findings provide compelling evidence that RNF145 regulates p70S6K activation through the turnover of PP2A subunit Aα, a key structural subunit of the protein phosphatase 2A (PP2A) complex.

### Re-expression of RNF145 restores PP2A-regulated p70S6K activation and ASM cell proliferation

**Figure 5:**
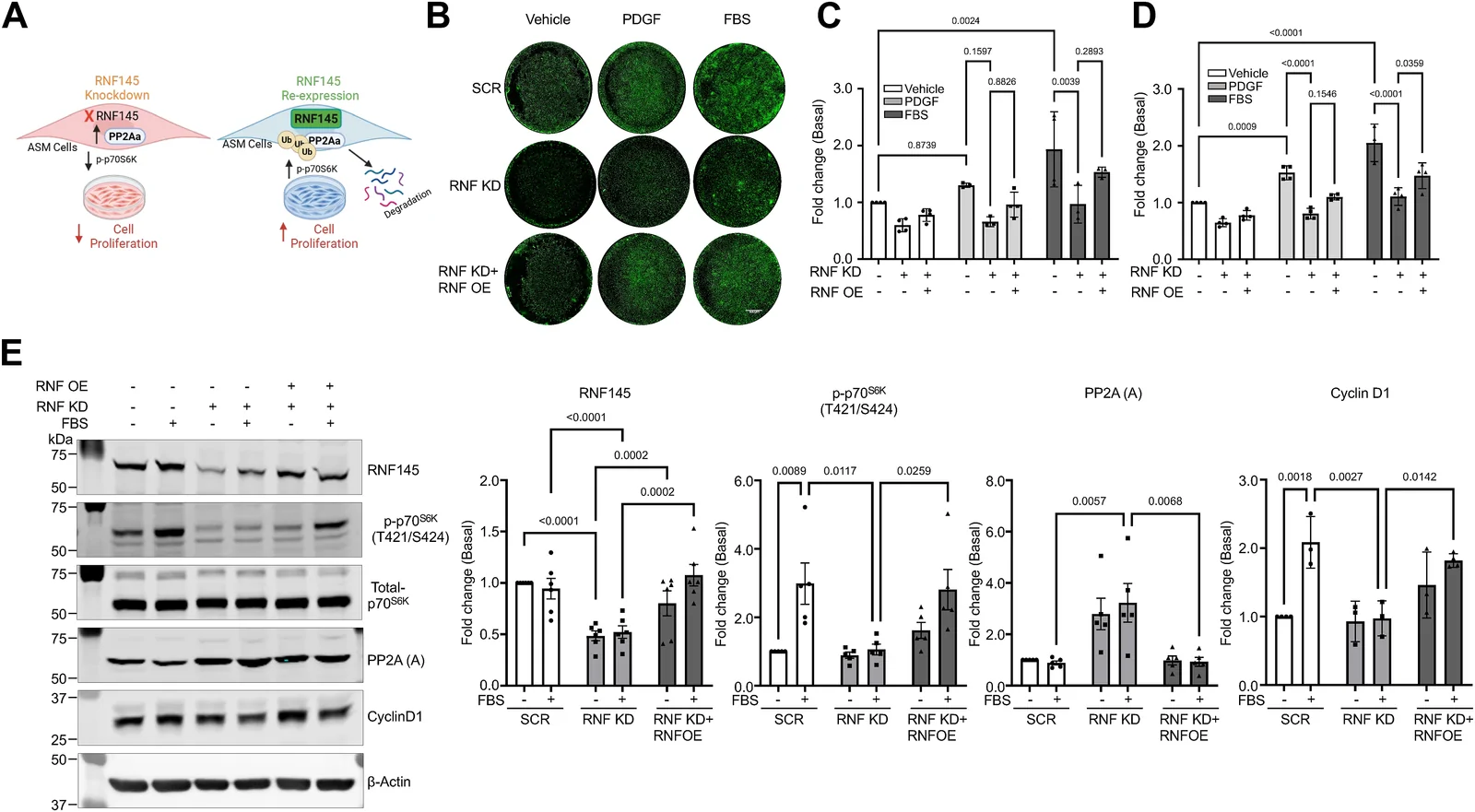
**Re-expression of RNF145 restores PP2A-regulated p70S6K activation and proliferation in RNF145-deficient ASM cells**. *A*, human ASM cells with stable RNF145 knockdown were re-expressed with RNF145 and then stimulated with PDGF (10 ng/ml) or FBS (10%) for 72 h. Cell proliferation was assessed using CyQuant and Alamar Blue assays. *B*, representative CyQuant fluorescence images. *C*, quantification of CyQuant fluorescence intensity as a measure of DNA content, normalized to basal conditions (mean ± SD, n = 4–6). *D*, alamar Blue fluorescence intensity normalized to basal conditions (mean ± SD, n = 4). *E*, representative immunoblots and quantitative densitometric analyses are shown. Statistical analyses were performed using One-way or two-way ANOVA followed by Tukey’s multiple-comparisons test. Exact *p* values are indicated in the figures. A *p* value < 0.05 was considered statistically significant.

To further confirm the role of RNF145 in ASM cell proliferation and pro-mitogenic signaling regulation, we reexpressed RNF145 in RNF145 knockdown cells (Fig. 5*A*). Silencing RNF145 significantly reduced mitogen-induced ASM cell proliferation compared with control cells. Notably, re-expression of RNF145 significantly restored mitogen-induced ASM cell proliferation (Fig. 5, *B*–*D*). Consistent with this effect, RNF145 re-expression also rescued phosphorylation of p70S6K at T421/S424 and restored cyclin D1 expression, both of which were markedly reduced in RNF145-deficient cells (Fig. 5*E*). Re-expression of RNF145 decreased protein levels of the PP2A scaffold subunit Aα (Fig. 5*E*). Together, these findings indicate that RNF145 promotes ASM cell proliferation by regulating PP2A-dependent p70S6K signaling.

### RNF145 regulates PP2A stability through a putative ubiquitination-dependent mechanism

**Figure 6:**
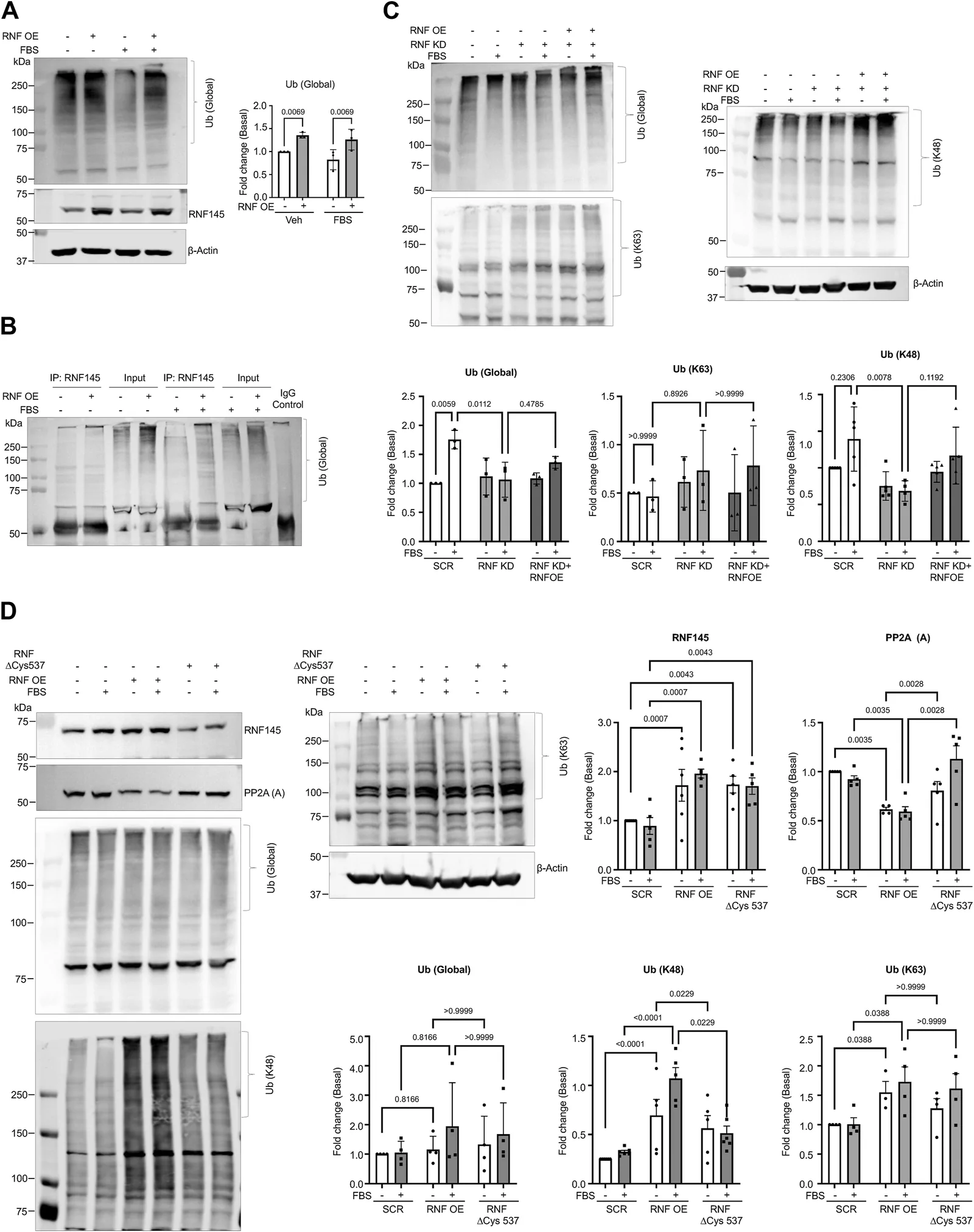
**RNF145 regulates PP2A stability through a putative ubiquitination-dependent mechanism.***A*, HEK293 cells were transfected with RNF145, and global ubiquitination was assessed by immunoblotting. Representative immunoblots and quantitative densitometric analyses are shown. *B*, RNF145 was immunoprecipitated from RNF145-overexpressing HEK293 cells, followed by immunoblotting for global ubiquitination. Representative immunoblots are shown. *C*, human ASM cells with stable RNF145 knockdown were re-expressed with RNF145 and then stimulated with FBS (10%) for 30 min. Global, K48-linked, and K63-linked ubiquitination were assessed by immunoblotting. Representative immunoblots and quantitative densitometric analyses are shown. *D*, human ASM cells were transfected with wild-type RNF145 or a ligase-deficient RING-domain mutant (C537 A). RNF145, PP2A subunit Aα, and global, K48-linked, and K63-linked ubiquitination were assessed by immunoblotting. Representative immunoblots and quantitative densitometric analyses are shown. Statistical analyses were performed using one-way or two-way ANOVA followed by Tukey’s or Bonferroni’s multiple-comparisons test, as appropriate. Exact *p* values are indicated in the figures. A *p* value < 0.05 was considered statistically significant.

Because RNF145 is a RING-type E3 ubiquitin ligase and its modulation altered PP2A subunit Aα protein levels, we investigated whether RNF145 regulates PP2A stability through ubiquitin-mediated proteostasis. Overexpression of RNF145 in HEK293 cells increased global ubiquitin conjugates (Fig. 6*A*). Immunoprecipitation of RNF145 followed by immunoblotting for ubiquitin revealed a marked increase in the global ubiquitination of RNF145-associated proteins under overexpression conditions (Fig. 6*B*). In ASM cells, RNF145 knockdown reduced both global and K48-linked polyubiquitin conjugates and re-expression of RNF145 restored them; K63-linked ubiquitination was unaffected (Fig. 6*C*). To test whether this effect required RNF145 E3 ligase activity, we expressed a RING-domain mutant RNF145 (C537A). Unlike wild-type RNF145, the C537A mutant failed to reduce PP2A subunit Aα protein levels (Fig. 6*D*). These results indicate that RNF145 regulates PP2A protein turnover through a K48-linked, RING domain-dependent ubiquitination mechanism.

## Discussion

ASM cell proliferation is orchestrated by a complex network of signaling pathways initiated by receptor tyrosine kinases (RTKs), G protein-coupled receptors (GPCRs), and mitogen-activated protein kinases (MAPKs) (33, 34). These pathways are activated by growth factors, cytokines, and inflammatory mediators, and their sustained activation contributes to airway remodeling in asthma and other chronic obstructive diseases. Given the clinical significance of airway remodeling, studies investigating molecular mediators of ASM proliferation are imperative and may pave the way for the discovery of new therapeutic targets. Various transcriptional, post-transcriptional, and translational mechanisms underlie ASM proliferation regulation. Post-translational mechanisms, such as ubiquitination, provide an additional layer of regulation for these proliferative signals. In this context, E3 ubiquitin ligases have recently attracted attention for their role in determining substrate specificity for ubiquitination and protein degradation.

RNF145, a RING-type E3 ubiquitin ligase, has been implicated in cholesterol biosynthesis and has also been associated with lung growth and function (19, 25). However, the molecular and cellular mechanisms by which RNF145 contributes to lung development and function remain unclear. In this study, we demonstrate that pharmacologic inhibition of RING E3 ligase with SMER3 or molecular knockdown of RNF145 suppresses mitogen-induced proliferation of human ASM cells (Fig. 1, *A*–*I*), suggesting the pro-mitogenic role of RNF145 in ASM cells. In consistent with our findings, RNF145’s role in the control of cell proliferation in several cancer models has been reported (20, 21, 22). Another study demonstrated that SMER3, a selective small-molecule inhibitor of RING E3 ligases, disrupts the interaction of Skp1-Cullin–F-box (SCF) complexes (35), which are central to cell proliferation (36). Our findings identify RNF145, another RING E3 ligase, that influences proliferative signaling in non-malignant airway-resident cells and, most importantly, position support further evaluation of RNF145 inhibition as a potential strategy for mitigating excessive ASM cell proliferation seen in asthma.

Growth factors, cytokines, chemokines, and GPCR agonists released by immune cells and resident airway cells regulate ASM cell proliferation (37, 38, 39). In this study, we employed FBS and PDGF, two well-established growth-promoting mediators. Although upstream signal transduction may vary among these mediators, ERK MAPK, PI3K-AKT, and mTOR pathways are predominant regulators of ASM cell proliferation (40, 41). Our findings demonstrate that RNF145 regulates the activity of p70S6K, a key downstream effector of AKT-mTOR signaling (Fig. 2, *A* and *B*). RNF145 inhibition suppressed p70S6K phosphorylation and reduced early cyclin D1 expression, consistent with impaired mitogenic signaling. Because cyclin D1 was measured at 30 min, whereas cell-cycle distribution was assessed at 24 h, these readouts likely represent distinct temporal consequences of RNF145 loss rather than a single checkpoint defect. Thus, although RNF145 deficiency was associated with G2/M accumulation at 24 h, the reduction in cyclin D1 is more appropriately interpreted as an early defect in proliferative signaling than as a direct explanation for the later cell-cycle profile. Notably, neither mTORC1 nor MAPK/ERK1/2 activation was altered by RNF145 perturbation, indicating that RNF145 regulates p70S6K through a mechanism independent of canonical upstream kinase activation.

We explored the detailed mechanism by which RNF145 regulates p70S6K activation. While activation of p70S6K is driven by upstream kinases within the AKT signaling pathway (42, 43), its inactivation is governed by serine-threonine protein phosphatases (PP1/PP2A) through dephosphorylation mechanisms (29, 30, 31, 32). Our findings suggest that RNF145 regulates p70S6K activity by modulating the activity of PP1 and PP2A phosphatases. This is supported by our observations that RNF145 inhibition increases PP1/PP2A activity and PP1 and PP2A phosphatase inhibitors such as OA and Cal-A rescue p70S6K phosphorylation.

Protein phosphatases are equally central to the control of signaling dynamics. Among these, serine/threonine phosphatase 2A (PP2A) plays a pivotal role in maintaining airway homeostasis by limiting pro-proliferative signaling and inflammation (44, 45). PP2A acts as a heterotrimeric complex composed of a catalytic (C), scaffold (A), and regulatory (B) subunit, with the Aα subunit being essential for holoenzyme stability and substrate recognition (46, 47). Loss or dysfunction of this structural subunit compromises PP2A activity, resulting in sustained activation of proliferation-promoting kinases such as p70S6K (48).

Our data support a model in which RNF145 negatively regulates PP2A function *via* its effect on the Aα subunit. RNF145 knockdown increased PP2A subunit Aα protein abundance, whereas RNF145 overexpression decreased it (Fig. 4, *A* and *B*). This reciprocal relationship implies that RNF145 promotes degradation of the PP2A structural subunit through ubiquitination and proteasomal targeting. Cycloheximide chase experiments further confirmed that RNF145 accelerates PP2A subunit Aα turnover, correlating with enhanced p70S6K phosphorylation and sustained mitogenic signaling. Restoration of p70S6K phosphorylation in RNF145-deficient cells by the phosphatase inhibitors OA and Cal-A supports this mechanism (Fig. 3*B*). Importantly, re-expression of RNF145 restored PP2A-regulated p70S6K activation and rescued proliferative responses in ASM cells, establishing RNF145 as a key regulator linking phosphatase turnover to downstream signaling and cell growth. Our data also support a ubiquitination-dependent mechanism for PP2A regulation. RNF145 contains a RING finger domain characteristic of E3 ubiquitin ligases, and mutation of the catalytic cysteine residue (C537 A), which disrupts ligase activity, abolished the ability of RNF145 to regulate PP2A expression (Fig. 6*D*). This finding indicates that the enzymatic activity of RNF145 is required for PP2A destabilization and supports a model in which RNF145-mediated ubiquitination targets PP2A components for proteasomal degradation. Although the precise ubiquitination sites remain to be identified, these results directly link RNF145 catalytic activity to the control of PP2A protein abundance. Future studies should delineate the specific ubiquitin linkage types catalyzed by RNF145 on PP2AAα and determine whether additional PP2A components or phosphatase regulators are similarly affected. Identifying the upstream cues that regulate RNF145 expression or activation under inflammatory or mechanical stress could also clarify its role in disease progression.

Ubiquitin-dependent control has emerged as an important mechanism for tuning mTOR pathway output, with multiple E3 ligases and deubiquitinases regulating the abundance, localization, and activity of key signaling components rather than simply switching kinase activity on or off (49). In this context, the proposed RNF145-dependent suppression of PP2A adds a distinct phosphatase-directed layer of regulation. The functional consequence of ubiquitination, however, depends not only on chain type but also on the site of ubiquitin attachment, as site-specific ubiquitination can influence substrate stability, unfolding, and proteasomal engagement, whereas K48-linked chains are classically associated with proteasomal degradation (50). Thus, identification of the ubiquitinated lysine residue(s) on PP2A subunit Aα will be important for defining the molecular basis of its turnover and for distinguishing ubiquitin marking from a structurally effective degradation signal (50).

Although the present data point most strongly to PP2A, other phosphatases may also contribute to regulation of the broader PI3K-Akt-mTOR-S6K axis in a context-dependent manner. Recent reviews of S6K biology note roles for both PP2A and PHLPP in S6K dephosphorylation, while earlier functional analyses support PP2A as a major S6K phosphatase in intact cells (51). This is particularly relevant from a translational perspective because direct pharmacologic targeting of phosphatases has historically been more difficult than kinase inhibition, owing in part to the conserved nature of phosphatase active sites and the challenge of achieving selectivity (50). Our findings therefore raise the possibility that phosphatase activity may instead be modulated indirectly through control of phosphatase stability or holoenzyme assembly by the ubiquitin system, providing an alternative strategy for restoring signaling balance in chronic airway disease (50).

**Figure 7:**
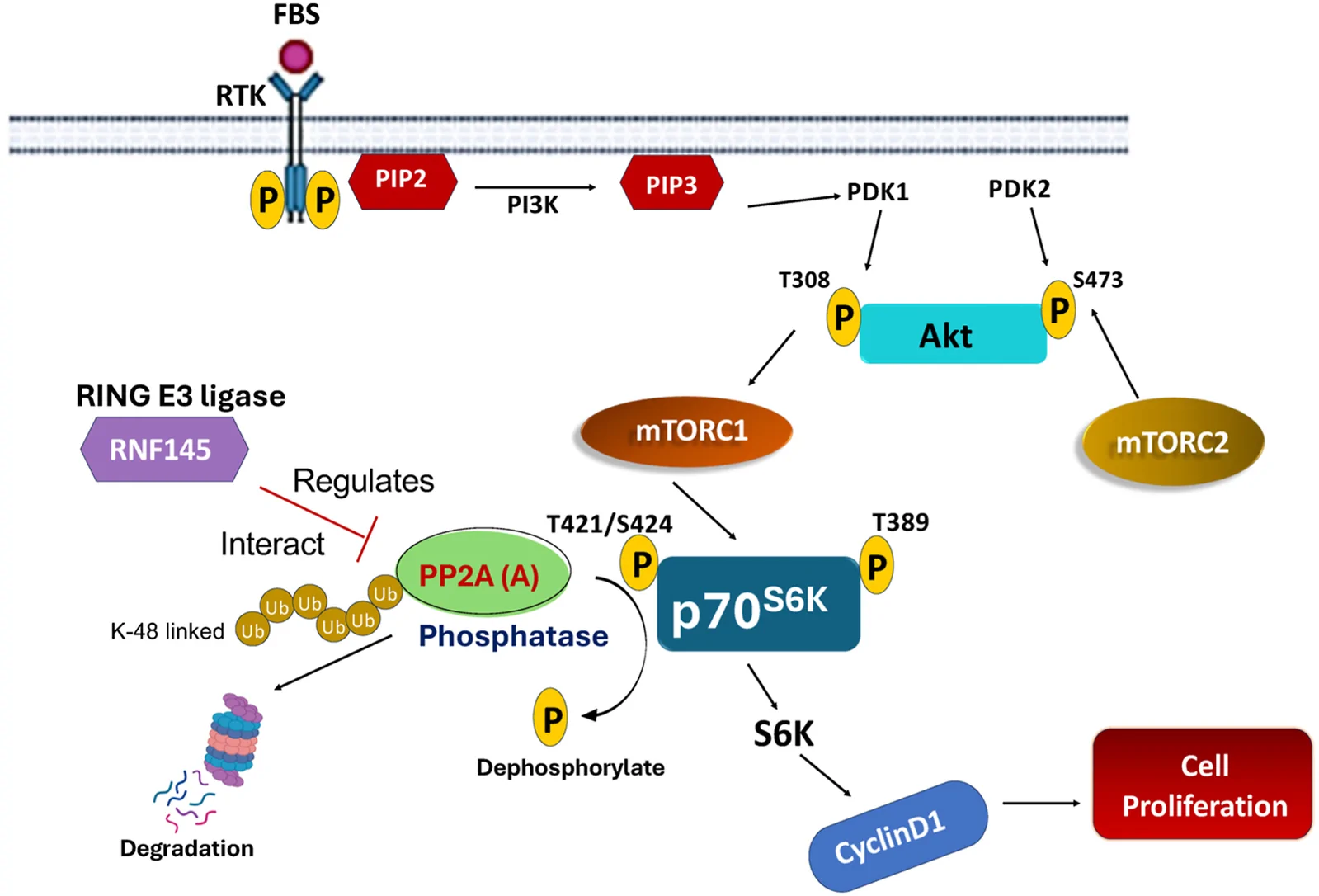
**RNF145 regulates ASM cell proliferation through ubiquitin-dependent control of the PP2A-p70S6K signaling axis**. Schematic model illustrating the proposed mechanism by which RNF145 modulates ASM cell proliferation. Activation of receptor tyrosine kinases (RTKs), G protein-coupled receptors (GPCRs), and mitogen-activated protein kinases (MAPKs) initiates proliferative signaling cascades involving PI3K-AKT-mTOR-p70S6K pathways. RNF145, a RING E3 ubiquitin ligase, promotes K-48 linked ubiquitin-dependent degradation of the PP2A scaffolding subunit Aα, thereby reducing PP2A holoenzyme activity. Loss of PP2A activity limits dephosphorylation of p70S6K at Thr421/Ser424, sustaining its activation and enhancing downstream induction of cyclin D1 and cell proliferation. Pharmacologic inhibition or knockdown of RNF145 stabilizes PP2A complexes, restoring phosphatase-mediated dephosphorylation of p70S6K and attenuating mitogen-induced ASM proliferation. This model defines RNF145 as a key post-translational regulator linking ubiquitin signaling to phosphatase control in airway remodeling.

In summary (Fig. 7), our findings identify RNF145 as a key regulator of ASM proliferation that sustains mTOR-p70S6K signaling through suppression of PP2A. By promoting K48-linked ubiquitination and proteasomal degradation of the PP2A scaffolding subunit Aα, RNF145 limits PP2A holoenzyme assembly and maintains proliferative signaling. This RNF145–PP2A axis reveals a previously unrecognized connection between ubiquitin-dependent proteostasis and airway smooth muscle growth control, with broader implications for mechanisms of airway remodeling in chronic lung disease.

## Experimental pprocedures

### Cell culture

Human Airway Smooth Muscle (ASM) cells were isolated from de-identified lung donors and cultured (passage 2–6) using complete F-12 medium supplemented with 10% FBS, penicillin/streptomycin, HEPES buffer, CaCl_2_, NaOH, and L-Glutamine (52). All cell cultures were replenished with fresh medium every 2 days. Cells were cultured until ∼80% confluency and then serum-starved in serum-free medium supplemented with 1% ITS (Gibco, #51500056) for 24 or 48 h before the experiment.

### Cell proliferation assays

Human ASM cells were plated in a black, clear-bottom 96-well plate at a density of 4000 cells per well and maintained in complete F-12 medium. After 24 h, cells were starved with serum-free F-12 medium and pre-treated with SMER3 (MCE, # HY-10949) (10, 30, 100, 300, 500, and 1000 nM) for 30 min, followed by 10 ng/ml PDGF (Cell Signaling Technology, #50611S) or 10% FBS for 72 h. Treatment with 0.1% DMSO (Sigma-Aldrich, #D2438) served as the vehicle control. After 72 h of treatment, the medium was aspirated and cells were incubated with CyQuant dye (Invitrogen, #C35006) or Alamar Blue (Invitrogen, #DAL1100) for 1 h at 37 ˚C. Fluorescence intensity was measured using FlexStation 3 (Molecular Devices, LLC) (Ex/Em at 480 nm/520 nm for CyQuant and 560 nm/590 nm for Alamar blue) and fluorescence images were captured using the GFP filter cube on an EVOS M700 imaging system (Thermo Fisher Scientific) with a 1.25 × objective. Data were normalized to vehicle-treated cells and expressed as fold change.

### Cell cycle analysis

Human ASM cells were cultured and treated with SMER3 as indicated in cell proliferation assay conditions, harvested by trypsinization, and washed twice with cold phosphate-buffered saline (PBS). Cells were then fixed in 70% ethanol added dropwise while vortexing and stored at −20 °C for at least 2 h. Following fixation, cells were washed twice with PBS to remove residual ethanol and resuspended in staining solution containing propidium iodide (50 μg/ml; Invitrogen, #BMS500PI) and RNase A (100 μg/ml; Invitrogen, #12091039) in PBS. Stained cells were analyzed using a flow cytometer (Cytek Aurora) and at least 10,000 events per sample were acquired. Doublets and cell debris were excluded by forward and side scatter gating and pulse processing (FSC-A vs. FSC-H). DNA content histograms were generated, and the percentage of cells in G0/G1, S, and G2/M phases was determined using FlowJo software version11 (BD Biosciences).

### Serine/threonine phosphatase assay

Serine/threonine phosphatase activity was measured using a phospho-peptide substrate (K-R-pT-I-R-R) kit (Sigma-Aldrich, #17–127). Human ASM cells were cultured and treated with SMER3 as indicated in cell proliferation assay conditions. Cells were washed and extracted in phosphatase extraction buffer containing 20 mM imidazole-HCl, 2 mM EDTA, 2 mM EGTA, pH 7.0 supplemented with protease inhibitors but without phosphatase inhibitors. Cells were sonicated for 10 s and centrifuged at 12,000×*g* for 10 min at 4 °C, followed by purification of endogenous phosphate using Sephadex G25 columns. Equal amounts of protein (typically 20–50 μg measured using BCA assay) were incubated with the phospho-peptide substrate (K-R-pT-I-R-R; final concentration 200 μM) in an assay buffer at 30 °C for 20 to 30 min. The reaction was terminated by the addition of malachite green reagent, and the release of free inorganic phosphate was quantified colorimetrically by measuring absorbance at 650 nm using a microplate reader. Phosphate release was calculated using a standard curve generated with known concentrations of inorganic phosphate and expressed as phosphatase activity (nmol Pi released/min/mg protein). Okadaic acid (PP2A/PP1 inhibitor)-treated cells were used as an inhibition control, and background controls lacking substrate or lysate were included to account for non-enzymatic hydrolysis.

### Lentiviral transductions and transfections

We obtained pLV-based short-hairpin constructs targeting RNF145 (VB230808–1371vgm), scrambled control (VB010000–0009mxc), RNF145 overexpression vector (VB241022-1387zvm) and RNF145 Δ Cys537 A vector denoted as C537 A (VB251118-1303gmp) from VectorBuilder. HEK293 T cells were transfected with pLV, lentiviral packaging plasmid (psPAX2) (Addgene, #12260) and VSV-G envelope plasmid (pMD2.G) (Addgene, #12259) using the PEI (Polyethyleneimine) transfection reagent (Polysciences, #19850) at a 1:3 (DNA: PEI) ratio. Virus-containing supernatants were collected 48 h of transfection, and the viral particles were concentrated using 10 kDa Amicon filters. Human ASM cells or HEK293 cells were transduced with the viral particles in the presence of 1 μg/ml polybrene (Sigma-Aldrich, #TR-1003). After 48 h of transduction, puromycin (2 μg/ml, Gibco, #A1113803) was added to select cells stably expressing RNF145 shRNA. For overexpression, RNF145 overexpression plasmid (2, 4, or 8 μg) was mixed with PEI (1:3 ratio) and transfected into HEK293 cells for 48 h. RNF knockdown and overexpression were confirmed by western blotting.

### Immunoblotting

Cells were lysed in RIPA buffer (Cell Signaling Technology, #9806S) supplemented with protease and phosphatase inhibitor (Selleck, #B14002 and #B15002) at 4 °C for 30 min. Lysates were then mixed with Laemmli buffer (Bio-Rad, #1610747) containing 10% β-mercaptoethanol (Sigma-Aldrich, #M6250) and boiled at 95 °C for 5 min 10 to 30 μg of samples were loaded onto 4 to 15% SDS-PAGE gels, and proteins were transferred to a nitrocellulose membrane. The membrane-bound proteins were blocked with 3% BSA (Fisher Scientific, #BP1605–100) in TBST for 1 h. Target proteins were detected by incubation with antigen-specific primary antibodies RNF145 (1:1000, #PA5-57718, Invitrogen), p-p70S6K (T421/S424, 1:1000, #9204, CST), p-p70S6K (T389, 1:1000, #9234, CST), p70S6K (1:1000, #9202, CST), p-AKT (T308, 1:1000, #2965, CST), p-AKT (S473, 1:1000, #4060, CST), AKT (1:1000, #9272, CST), Cyclin D1 (1:1000, #55506, CST), p-mTOR (Ser2448, 1:1000, #5536, CST), p-mTOR (Ser2481, 1:1000, #2974, CST), mTOR (1:1000, #2972, CST), p-ERK1/2 (1:1000, #4370, CST), ERK1/2 (1:1000, #4695, CST), PP2A subunit Aα (1:1000, #2041, CST), PP2A subunit B (1:1000, #2290, CST), PP2A subunit C (1:1000, #2259, CST), Anti-Ubiquitin (Global 1:1000, #ab134953, Abcam), Anti-Ubiquitin (K48, 1:1000 #ab140601, Abcam), Anti-Ubiquitin (K63, 1:1000, #ab179434, Abcam), β-actin (1:50,000, Sigma) overnight in 3% BSA in TBST. Goat anti-rabbit IR 800 or Goat anti-mouse IR 680 secondary antibodies (LICOR Biosciences, #926–32232 and #926–68078, 1:10,000 in 3% BSA in TBST) were used to detect the target protein using the Odyssey infrared scanner (LI-COR Biosciences). Band intensities were quantified using LI-COR Image Studio Lite software version 5.2 (LI-COR Biosciences) and Image J 1.54g (National Institute of Health). Densitometric values for each target protein were normalized to the corresponding loading control (β-actin). For phospho-protein analyses, phospho-protein signals were normalized to either the corresponding total protein or loading control as indicated. Fold-change values relative to the control group were used for graphical presentation. Individual data points and exact *p* values are provided in the figures. Statistical analyses were performed using these normalized values in GraphPad Prism. Data are presented as mean ± SD from independent biological replicates. Statistical significance was determined using one-way or two-way ANOVA followed by the appropriate *post hoc* test. A value of *p* < 0.05 was considered statistically significant.

### Immunoprecipitation

The lysates were incubated overnight with the appropriate antibody at 4 °C. Protein A-Magnetic beads (MCE, #HY-K0202) were added, and the samples were mixed for an additional 1 h at room temperature. The beads were washed four times with PBST. The samples were boiled for 5 min at 70 °C in 1X SDS sample buffer, and the immune complexes were separated by magnetic stand.

### Statistical analysis

Data are presented as mean ± SD. Statistical analyses were performed using GraphPad Prism version 10.3.0 (GraphPad Software, Boston, MA, USA). Groups were compared using one-way or two-way ANOVA, followed by Tukey’s, Bonferroni’s, or Šídák’s multiple-comparisons test, as appropriate. Exact *p* values are reported in the corresponding figures. A *p* value < 0.05 was considered statistically significant.

## Data availability

The data supporting the findings of this study are available from the corresponding author upon reasonable request.

## Conflict of interest

The authors declare that they have no conflicts of interest with the contents of this article.

## References

[1] Amrani Y., Panettieri R.A. (2003). Airway smooth muscle: contraction and beyond. Int. J. Biochem. Cell Biol..

[2] Johnson P.R., Roth M., Tamm M., Hughes M., Ge Q., King G. (2001). Airway smooth muscle cell proliferation is increased in asthma. Am. J. Respir. Crit. Care Med..

[3] Barnes P.J. (1998). Pharmacology of airway smooth muscle. Am. J. Respir. Crit. Care Med..

[4] Hirota J.A., Nguyen T.T., Schaafsma D., Sharma P., Tran T. (2009). Airway smooth muscle in asthma: phenotype plasticity and function. Pulm. Pharmacol. Ther..

[5] Fang L., Sun Q., Roth M. (2020). Immunologic and non-immunologic mechanisms leading to airway remodeling in asthma. Int. J. Mol. Sci..

[6] Burton J.C., Grimsey N.J. (2019). Ubiquitination as a key regulator of endosomal signaling by GPCRs. Front Cell Dev Biol.

[7] Haglund K., Dikic I. (2005). Ubiquitylation and cell signaling. EMBO J..

[8] Tai H.C., Schuman E.M. (2008). Ubiquitin, the proteasome and protein degradation in neuronal function and dysfunction. Nat. Rev. Neurosci..

[9] Schwertman P., Bekker-Jensen S., Mailand N. (2016). Regulation of DNA double-strand break repair by ubiquitin and ubiquitin-like modifiers. Nat. Rev. Mol. Cell Biol..

[10] Buetow L., Huang D.T. (2016). Structural insights into the catalysis and regulation of E3 ubiquitin ligases. Nat. Rev. Mol. Cell Biol..

[11] Yang Q., Zhao J., Chen D., Wang Y. (2021). E3 ubiquitin ligases: styles, structures and functions. Mol. Biomed..

[12] Bulatov E., Ciulli A. (2015). Targeting Cullin-RING E3 ubiquitin ligases for drug discovery: structure, assembly and small-molecule modulation. Biochem. J..

[13] Jeong Y., Oh A.R., Jung Y.H., Gi H., Kim Y.U., Kim K. (2023). Targeting E3 ubiquitin ligases and their adaptors as a therapeutic strategy for metabolic diseases. Exp. Mol. Med..

[14] Ju L.G., Zhu Y., Long Q.Y., Li X.J., Lin X., Tang S.B. (2019). SPOP suppresses prostate cancer through regulation of CYCLIN E1 stability. Cell Death Differ..

[15] Li C., Du L., Ren Y., Liu X., Jiao Q., Cui D. (2019). SKP2 promotes breast cancer tumorigenesis and radiation tolerance through PDCD4 ubiquitination. J. Exp. Clin. Cancer Res..

[16] Mihashi Y., Mizoguchi M., Takamatsu Y., Ishitsuka K., Iwasaki H., Koga M. (2017). C-MYC and its main ubiquitin ligase, FBXW7, influence cell proliferation and prognosis in adult T-cell leukemia/lymphoma. Am. J. Surg. Pathol..

[17] Schrock M.S., Stromberg B.R., Scarberry L., Summers M.K. (2020). APC/C ubiquitin ligase: functions and mechanisms in tumorigenesis. Semin. Cancer Biol..

[18] Jiang L.Y., Jiang W., Tian N., Xiong Y.N., Liu J., Wei J. (2018). Ring finger protein 145 (RNF145) is a ubiquitin ligase for sterol-induced degradation of HMG-CoA reductase. J. Biol. Chem..

[19] Zhang L., Rajbhandari P., Priest C., Sandhu J., Wu X., Temel R. (2017). Inhibition of cholesterol biosynthesis through RNF145-dependent ubiquitination of SCAP. Elife.

[20] Rong L., Chen B., Liu K., Liu B., He X., Liu J. (2022). CircZDBF2 up-regulates RNF145 by ceRNA model and recruits CEBPB to accelerate oral squamous cell carcinoma progression via NFkappaB signaling pathway. J. Transl. Med..

[21] Xie N., Mei S., Dai C., Chen W. (2024). Palmdelphin inhibits ovarian cancer cell stem specification via downregulating ring finger protein 145. Crit. Rev. Eukaryot. Gene Expr..

[22] Li K., Yang Y., Ma M., Lu S., Li J. (2023). Hypoxia-based classification and prognostic signature for clinical management of hepatocellular carcinoma. World J. Surg. Oncol..

[23] Chen H., Liu J., Zhang J., Chen Y., Wang Y., Qiu Y. (2025). USP22/BRD4 mediated hedgehog pathway activation contributes to airway remodeling in asthma. Int. Immunopharmacol.

[24] Deng S., Ding L., Qian Y., Huang X. (2025). The role of ubiquitin-proteasome system (UPS) in asthma pathology. J. Asthma Allergy.

[25] Gorlanova O., Usemann J., Latzin P., Schmidt A., Michel S., Toncheva A. (2015). LATE-BREAKING ABSTRACT: genome-wide association studies identify novel loci associated with early postnatal lung function. Eur. Respir. J..

[26] Hernandez-Lara M.A., Yadav S.K., Conaway S., Shah S.D., Penn R.B., Deshpande D.A. (2023). Crosstalk between diacylglycerol kinase and protein kinase A in the regulation of airway smooth muscle cell proliferation. Respir. Res..

[27] Sharma P., P A., Pera T., Tiegs B.C., Hershfeld A., Kenyon L.C. (2016). Antimitogenic effect of bitter taste receptor agonists on airway smooth muscle cells. Am. J. Physiol. Lung Cell Mol. Physiol..

[28] Morgan S.J., Deshpande D.A., Tiegs B.C., Misior A.M., Yan H., Hershfeld A.V. (2014). beta-Agonist-mediated relaxation of airway smooth muscle is protein kinase A-dependent. J. Biol. Chem..

[29] Westphal R.S., Coffee R.L., Marotta A., Pelech S.L., Wadzinski B.E. (1999). Identification of kinase-phosphatase signaling modules composed of p70 S6 kinase-protein phosphatase 2A (PP2A) and p21-activated kinase-PP2A. J. Biol. Chem..

[30] Peterson R.T., Desai B.N., Hardwick J.S., Schreiber S.L. (1999). Protein phosphatase 2A interacts with the 70-kDa S6 kinase and is activated by inhibition of FKBP12-rapamycinassociated protein. Proc. Natl. Acad. Sci. U. S. A..

[31] Matsumoto N., Miyamoto Y., Hattori K., Ito A., Harada H., Oizumi H. (2020). PP1C and PP2A are p70S6K phosphatases whose inhibition ameliorates HLD12-Associated inhibition of oligodendroglial cell morphological differentiation. Biomedicines.

[32] Hsiao K.C., Ruan S.Y., Chen S.M., Lai T.Y., Chan R.H., Zhang Y.M. (2023). The B56gamma3-containing protein phosphatase 2A attenuates p70S6K-mediated negative feedback loop to enhance AKT-facilitated epithelial-mesenchymal transition in colorectal cancer. Cell Commun Signal..

[33] Krymskaya V.P., Orsini M.J., Eszterhas A.J., Brodbeck K.C., Benovic J.L., Panettieri R.A. (2000). Mechanisms of proliferation synergy by receptor tyrosine kinase and G protein-coupled receptor activation in human airway smooth muscle. Am. J. Respir. Cell Mol. Biol..

[34] Penn R.B., Benovic J.L. (2008). Regulation of heterotrimeric G protein signaling in airway smooth muscle. Proc. Am. Thorac. Soc..

[35] Aghajan M., Jonai N., Flick K., Fu F., Luo M., Cai X. (2010). Chemical genetics screen for enhancers of rapamycin identifies a specific inhibitor of an SCF family E3 ubiquitin ligase. Nat. Biotechnol..

[36] Petroski M.D., Deshaies R.J. (2005). Function and regulation of cullin-RING ubiquitin ligases. Nat. Rev. Mol. Cell Biol..

[37] Stamatiou R., Paraskeva E., Gourgoulianis K., Molyvdas P.A., Hatziefthimiou A. (2012). Cytokines and growth factors promote airway smooth muscle cell proliferation. ISRN Inflamm..

[38] Chen G., Khalil N. (2006). TGF-beta1 increases proliferation of airway smooth muscle cells by phosphorylation of map kinases. Respir. Res..

[39] Bosse Y., Thompson C., Audette K., Stankova J., Rola-Pleszczynski M. (2008). Interleukin-4 and interleukin-13 enhance human bronchial smooth muscle cell proliferation. Int. Arch. Allergy Immunol..

[40] Ma B., Athari S.S., Mehrabi Nasab E., Zhao L. (2021). PI3K/AKT/mTOR and TLR4/MyD88/NF-kappaB signaling inhibitors attenuate pathological mechanisms of allergic asthma. Inflammation.

[41] Yap H.M., Israf D.A., Harith H.H., Tham C.L., Sulaiman M.R. (2019). Crosstalk between signaling pathways involved in the regulation of airway smooth muscle cell hyperplasia. Front Pharmacol..

[42] Sunami T., Byrne N., Diehl R.E., Funabashi K., Hall D.L., Ikuta M. (2010). Structural basis of human p70 ribosomal S6 kinase-1 regulation by activation loop phosphorylation. J. Biol. Chem..

[43] Pullen N., Thomas G. (1997). The modular phosphorylation and activation of p70s6k. FEBS Lett..

[44] Hunter T. (1995). Protein kinases and phosphatases: the yin and yang of protein phosphorylation and signaling. Cell.

[45] Reza M.I., Kumar A., Pabelick C.M., Britt R.D., Prakash Y.S., Sathish V. (2024). Downregulation of protein phosphatase 2Aalpha in asthmatic airway smooth muscle. Am. J. Physiol. Lung Cell Mol. Physiol..

[46] Haanen T.J., O'Connor C.M., Narla G. (2022). Biased holoenzyme assembly of protein phosphatase 2A (PP2A): from cancer to small molecules. J. Biol. Chem..

[47] Lubbers E.R., Mohler P.J. (2016). Roles and regulation of protein phosphatase 2A (PP2A) in the heart. J. Mol. Cell Cardiol..

[48] Xie F., Bao X., Yu J., Chen W., Wang L., Zhang Z. (2015). Disruption and inactivation of the PP2A complex promotes the proliferation and angiogenesis of hemangioma endothelial cells through activating AKT and ERK. Oncotarget.

[49] Jiang Y., Su S., Zhang Y., Qian J., Liu P. (2019). Control of mTOR signaling by ubiquitin. Oncogene.

[50] Carroll E.C., Marqusee S. (2022). Site-specific ubiquitination: deconstructing the degradation tag. Curr. Opin. Struct. Biol..

[51] Wu X., Xie W., Xie W., Wei W., Guo J. (2022). Beyond controlling cell size: functional analyses of S6K in tumorigenesis. Cell Death Dis.

[52] Panettieri R.A., Murray R.K., DePalo L.R., Yadvish P.A., Kotlikoff M.I. (1989). A human airway smooth muscle cell line that retains physiological responsiveness. Am. J. Physiol..

